# Impact of Brain-Derived Neurotrophic Factor Val66Met Polymorphism on Cortical Thickness and Voxel-Based Morphometry in Healthy Chinese Young Adults

**DOI:** 10.1371/journal.pone.0037777

**Published:** 2012-06-13

**Authors:** Xuejuan Yang, Peng Liu, Jinbo Sun, Guihong Wang, Fang Zeng, Kai Yuan, Jixin Liu, Minghao Dong, Karen M. von Deneen, Wei Qin, Jie Tian

**Affiliations:** 1 Life Sciences Research Center, School of Life Sciences and Technology, Xidian University, Xi’an, Shaanxi, China; 2 The 3rd Teaching Hospital, Chengdu University of Traditional Chinese Medicine, Chengdu, Sichuan, China; 3 Institute of Automation, Chinese Academy of Sciences, Beijing, China; Cuban Neuroscience Center, Cuba

## Abstract

**Background:**

Following voxel-based morphometry (VBM), brain-derived neurotrophic factor (BDNF) Val66Met polymorphism (rs6265) has been shown to affect human brain morphology in Caucasians. However, little is known about the specific role of the Met/Met genotype on brain structure. Moreover, the relationship between BDNF Val66Met polymorphism and Chinese brain morphology has not been studied.

**Methodology/Principal Findings:**

The present study investigated brain structural differences among three genotypes of BDNF (rs6265) for the first time in healthy young Chinese adults via cortical thickness analysis and VBM. Brain differences in Met carriers using another grouping method (combining Val/Met and Met/Met genotypes into a group of Met carriers as in most previous studies) were also investigated using VBM. Dual-approach analysis revealed less gray matter (GM) in the frontal, temporal, cingulate and insular cortices in the Met/Met group compared with the Val/Val group (corrected, *P*<0.05). Areas with less GM in the Val/Met group were included in the Met/Met group. VBM differences in Met carriers were only found in the middle cingulate cortex.

**Conclusions/Significance:**

The current results indicated a unique pattern of brain morphologic differences caused by BDNF (rs6265) in young Chinese adults, in which the Met/Met genotype markedly affected the frontal, temporal, cingulate, and insular regions. The grouping method with Met carriers was not suitable to detect the genetic effect of BDNF Val66Met polymorphism on brain morphology, at least in the Chinese population, because it may hide some specific roles of Met/Met and Val/Met genotypes on brain structure.

## Introduction

With the rise of imaging genomics, more and more studies have demonstrated that brain structure and function are under strong genetic control [1]. As a strong candidate gene that could influence brain development and function, brain-derived neurotrophic factor (BDNF) is involved in regulating neuronal survival, proliferation and differentiation, as well as synaptic plasticity processes [2], [3]. A common single nucleotide polymorphism in the human BDNF gene (rs6265) leading to a substitution of methionine (Met) for valine (Val) at codon 66 in the pro-region of the BDNF protein (Val66Met) could result in disruption of activity-dependent BDNF secretion [4], [5]. In recent years, structural magnetic resonance imaging (MRI) scans have revealed that the BDNF Val66Met polymorphism can influence human brain morphology. The Met allele carriers showed less gray matter (GM) volume in some brain areas, such as the hippocampus [6], [7], subgenual anterior cingulated [8], dorsolateral prefrontal cortex (DLPFC) [7], [9], amygdale [10], and temporal and occipital lobar regions [11], compared with Val/Val homozygotes. However, these previous studies have provided little information on individuals who are homozygous for the Met allele (Met/Met) because of its small frequency in Caucasians. The Met/Met genotype is rare and is found only about 4% (Met allele frequency: 20% to 30%) of the Caucasian populations [4], [12], which results in rigorous demand on sample size when obtaining information on only Met homozygotes in this race. As a result, the Met/Met homozygotes and the Val/Met heterozygotes were often merged into a group of Met carriers for analyses in most of these studies, which undoubtedly limited the detection of the specific role of the Met/Met genotype on brain structure.

Compared to the Caucasian populations, Asians have a higher Met allele frequency, namely more than 40% [12], [13]. In addition, 20–30% of the Met/Met homozygote distribution and nearly 50% of the Met allele frequency exist in the Chinese population [14], [15], which allows analysis of the Met/Met genotype alone to be conducted more readily. To date, data with regards to the relationship between BDNF (rs6265) genotypes and brain structure in the Chinese population is lacking. Moreover, some physiological processes related to BDNF (rs6265) have already been linked racial differences, such as mental diseases in which an association between the Val66Met polymorphism and schizophrenia exists in Scottish, Spanish and American individuals [16], [17], [18], unlike the Chinese population [14]. Thus, whether or not the influence of the genetic variant of BDNF (rs6265) on brain anatomy also varies among races is worth discussion. A large-scale general population study on this problem is necessary.

In addition, to our knowledge (online search in PubMed, http://www.ncbi.nlm.nih. gov/pubmed ), most studies measuring structural brain differences among BDNF(rs6265) genotypes were based on GM volume using voxel-based morphometry (VBM). VBM is a good automatic method to assess volumetric differences over the whole brain. This method offers information on GM volume from cortical and subcortical areas by combining all structure properties (thickness, surface area, and folding), however, these properties are essentially independent from each other. Compared with VBM, the method of cortical thickness measurement provides a direct examination on thickness independent of surface or position variance, and allows for better matching of homologous cortical regions using geometry to do inter-subject registration [19], [20], [21]. If these two methods are used toghther, the results driven by dual-analysis would be more credible [22], [23]. However, few studies have focused on the relevance of cortical thickness to the BDNF Val66Met polymorphism. Despite a study reporting no relevance of cortical thickness with the rs6265 genotypes [24], a few recent studies have shown that BDNF (rs6265) contributed to the cortical thickness changes in the entorhinal cortex, temporal gyri and DLPFC [25], [26] in people at risk of developing Alzheimer’s disease or suffering from trauma. However, it remains unclear whether the cortical thickness in other brain areas was also affected by the BDNF Val66Met polymorphism in healthy subjects.

Therefore, in order to understand the impact of different genotypes of BDNF Val66Met polymorphism on brain structural plasticity, the present study combined for the first time cortical thickness analysis and VBM to investigate the structural brain differences among three BDNF Val66Met genotypes (Val/Val, Val/Met and Met/Met) in healthy young Chinese adults. Moreover, we also observed differences in brain anatomy in Met allele carriers by merging the Val/Met with Met/Met groups, as performed in previous studies, to evaluate the differences caused by grouping methods.

## Results

### Allele and Genotype Distribution

Thirteen individuals did not participate in the MRI scanning because of quitting, and the MRI data of 7 participants was discarded because of unsuccessful DNA sequencing or MRI scanning. Therefore, 61 subjects in total were finally grouped according to their BDNF genotype, into 15 homozygous carriers of the Val allele (Val/Val), 17 homozygous carriers of the Met allele (Met/Met), and 29 heterozygous (Val/Met). The frequency of the BDNF genotypes was 24.6% for Val/Val, 47.5% for Val/Met, and 27.9% for Met/Met in our study of sample (Table 1), which was similar to previously published results based on the Chinese population [14], [27], [28]. The distribution of genotypes in healthy subjects was consistent with the Hardy–Weinberg Equilibrium (χ^2^ = 0.141, *P = *0.707). There were no significant differences between males and females with respect to genotype (χ^2^ = 4.077, *P = *0.13) and allele distribution (χ^2^ = 3.175, *P = *0.075).

**Table 1:** Genotype and allele frequencies of the BDNF Val66Met polymorphism in healthy young Chinese adults.

		Genotype (%)			Allele (%)	
		Val/Val	Val/Met	Met/Met	Val	Met
**Male**	n = 34	5	18	11	28	40
**Female**	n = 27	10	11	6	31	23
**Total**	n = 61	15(24.6)	29(47.5)	17(27.9)	59(48.4)	63(51.6)

### Cortical Thickness Differences between Val/Met, Met/Met and Val/Val Genotypes

**Figure 1:**
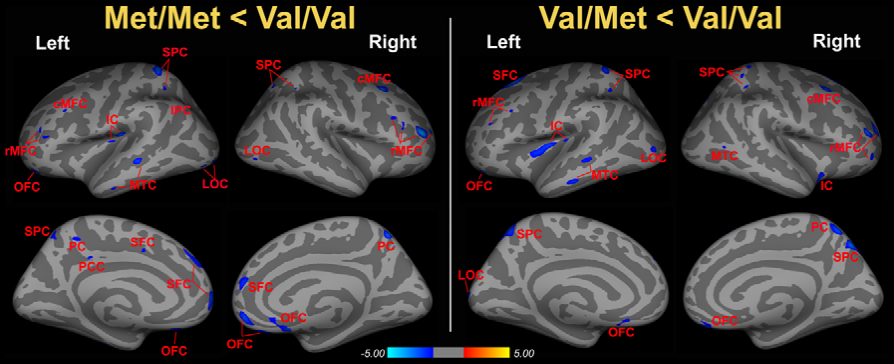
Cortical thickness differences among three genotypes based on the BDNF Val66Met polymorphism. Left panel: areas with a significantly thinning cortex in the Met/Met group compared with the Val/Val group (Met/Met < Val/Val, corrected, *P*<0.05). Right panel: areas with a significantly thinning cortex in the Val/Met group compared with the Val/Val group (Val/Met < Val/Val, corrected, *P*<0.05). cMFC, caudal middle frontal cortex; IC, insular cortex; IPC, inferior parietal cortex; LOC, lateral occipital cortex; MTC, middle temporal cortex; OFC, orbital frontal cortex; PC, precuneus cortex; PCC, posterior cingulate cortex; rMFC, rostral middle frontal cortex; SFC, superior frontal cortex; SPC, superior parietal cortex;. See Table 2 and Table 3 for details.

In general, compared to homozygous Val carriers there were no brain regions showing thickening in homozygous Met or heterozygous Met carriers. Compared with the Val/Val group, both the Met/Met and Val/Met groups exhibited a significantly thinner cortex in the frontal (superior, middle and orbital parts) cortex, middle temporal cortex (MTC), superior parietal cortex (SPC), lateral occipital cortex (LOC), insular cortex (IC) and precuneus cortex (PC) in one or both hemispheres (FDR corrected, *P*<0.05; Figure 1, Table 2 and 3). Moreover, the Met/Met group was observed to have reduced thickness in other areas, including the left inferior parietal cortex (IPC), and the posterior cingulate cortex (PCC) (FDR corrected, *P*<0.05; Figure 1 and Table 2).

**Table 2:** Areas with decreased cortical thickness in the Met/Met group compared with the Val/Val group based on the BDNF Val66Met polymorphism (FDR corrected, P<0.05).

Brain area	Side	Talariach coordinate					Size-mm^2^	Max^a^
		x		y	z			
Superior frontal cortex	Left	−2.37	61.6			4.7	2202.95	−2.38
	Left	−8.3	52.2			36.7	271.38	−2.17
	Left	−11.3	5.2			39.1	11.0	−1.37
Rostral middle frontal cortex	Left	−38.5	40.2			17.8	26.40	–1.37
	Left	−34.9	33.9			10.4	32.09	−1.43
Caudal middle frontal cortex	Left	−43.1	21.4			32.8	20.57	−1.53
Orbitofrontal cortex	Left	−8.8	31.5			−21.3	53.68	−1.75
	Left	−23.3	42.2			−10.7	80.20	−1.67
Superior parietal cortex	Left	−22.8	−48.1			58.1	236.11	−2.68
	Left	−8.7	−64.0			55.0	75.65	−1.56
	Left	−33.5	−68.3			43.9	23.72	−1.48
Inferior parietal cortex	Left	−33.5	−68.3			43.9	23.72	−1.48
Middle temporal cortex	Left	−49.1	−35.0			−8.4	68.74	−2.27
	Left	−53.9	−13.2			−24.9	22.81	−1.46
Posterior cingulate cortex	Left	−4.9	−33.7			38.7	13.03	−1.54
Lateral occipital cortex	Left	−25.9	−96.2			−14.6	56.22	−1.59
	Left	−37.6	−85.6			−15.3	27.26	−1.53
Precuneus cortex	Left	−10.4	−45.7			48.9	35.26	−1.45
Insular cortex	Left	−38.0	−24.1			20.6	94.32	−2.82
	Left	−33.3	−21.6			10.4	34.41	−1.54
Superior frontal cortex	Right	8.7	48.7			17.7	119.56	−2.01
Rostral middle frontal cortex	Right	34.8	48.6			14.3	260.90	−4.00
	Right	41.5	23.1			27.2	29.36	−1.45
	Right	22.9	53.6			15.2	30.18	−1.89
	Right	45.3	32.0			22.0	29.56	−1.42
Caudal middle frontal cortex	Right	32.7	16.9			47.7	138.38	−3.11
Orbitofrontal cortex	Right	7.5	14.0			−15.9	303.89	−2.50
	Right	10.8	49.5			−10.4	180.46	−2.49
	Right	13.8	39.5			−24.2	232.69	−3.05
Superior parietal cortex	Right	22.2	−61.2			50.4	25.32	−1.50
	Right	32.6	−45.7			44.0	11.52	−1.42
Lateral occipital cortex	Right	43.8	−80.9			−7.9	21.10	−1.48
Precuneus cortex	Right	6.0	−62.5			55.3	89.81	−2.85

**Table 3:** Areas with decreased cortical thickness in the Val/Met group compared with the Val/Val group based on the BDNF Val66Met polymorphism (FDR corrected, P<0.05).

Brain area	Side	Talariach coordinate					Size-mm^2^	Max^a^
		x		y	z			
Superior frontal cortex	Left	−21.8	21.5			55.8	416.98	−2.97
Rostral middle frontal cortex	Left	−43.5	22.0			32.5	20.77	−1.76
	Left	−40.9	38.1			22.9	15.01	−1.39
Orbitofrontal cortex	Left	−22.2	43.5			−12.7	56.96	−1.57
	Left	−6.4	28.9			−18.1	46.48	−1.55
Superior parietal cortex	Left	−24.7	−48.3			59.0	289.68	−2.35
	Left	−9.9	−62.2			55.2	306.25	−2.19
	Left	−28.9	−48.4			40.6	29.69	−1.67
Middle temporal cortex	Left	−50.0	−38.3			−6.8	67.79	−1.68
	Left	−57.4	−22.1			−20.3	82.84	−2.00
Lateral occipital cortex	Left	−37.8	−83.5			2.1	97.11	−1.89
	Left	−9.8	−102.1			4.1	104.15	−1.88
Insular cortex	Left	−31.9	−28.4			9.4	21.99	−1.64
	Left	−35.5	−12.3			7.6	266.69	−2.20
Rostral middle frontal cortex	Right	35.8	51.5			−4.4	57.83	−1.54
	Right	35.7	48.7			16.1	137.93	−3.02
	Right	22.8	52.4			16.2	137.47	−2.49
Caudal middle frontal cortex	Right	33.4	15.5			49.5	117.14	−2.62
Orbitofrontal cortex	Right	13.0	44.2			−23.7	237.25	−2.88
Superior parietal cortex	Right	19.7	−41.6			60.8	43.65	−1.74
	Right	33.5	−46.6			62.7	28.31	−1.56
	Right	32.3	−43.7			48.4	28.03	−1.44
	Right	9.9	−73.3			44.5	389.99	−2.98
Middle temporal cortex	Right	47.9	−61.5			4.5	12.65	−1.54
Precuneus cortex	Right	6.4	−61.5			56.5	145.21	−2.59
Insular cortex	Right	37.4	3.0			−15.9	118.57	−1.97

### VBM Differences between Val/Met, Met/Met and Val/Val Genotypes

Although numerous studies have investigated the relevance between the BDNF Val66Met polymorphism and GM volume, no study has focused on the Chinese population. In this study we observed GM volume differences among BDNF (rs6265) genotypes in young Chinese adults.

**Figure 2:**
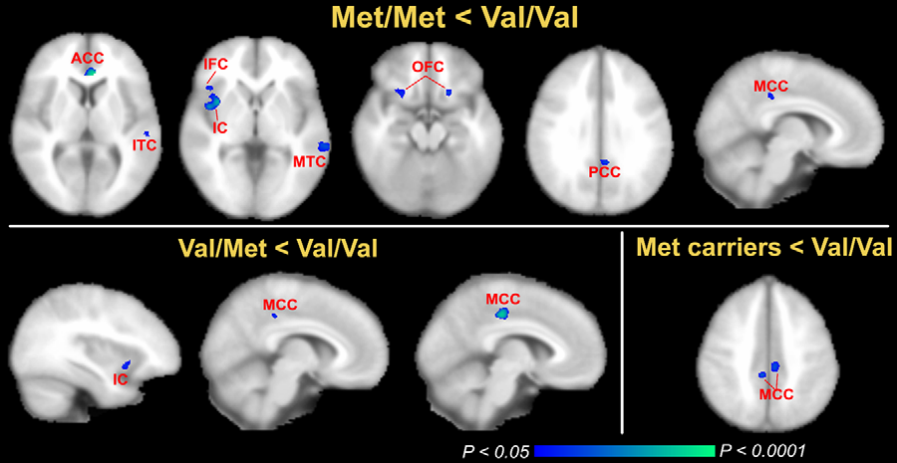
VBM differences among three genotypes based on the BDNF Val66Met polymorphism. Top panel: areas with a significantly decreased GM volume in Met/Met group compared with Val/Val group (Met/Met < Val/Val, corrected, *P*<0.05). Bottom left panel: areas with a significantly decreased GM volume in the Val/Met group compared with the Val/Val group (Val/Met < Val/Val, corrected, *P*<0.05). Bottom left panel: areas with a significantly decreased GM volume in the Met carriers group (Met/Met+Val/Met) compared with the Val/Val group (Met carriers < Val/Val, corrected, *P*<0.05 ). ACC, anterior cingulate cortex; MCC, middle cingulated cortex; MTC, middle temporal cortex; IC, insular cortex; IFC, inferior frontal cortex; OFC, orbital frontal cortex; PCC, posterior cingulate cortex; STC, superior temporal cortex.

No brain regions showed increased GM volume in the Met variant, and the total GM volume over the entire brain was not different among groups. Compared to the Val/Val group, Met/Met individuals had a smaller volume in some areas of the frontal, temporal, cingulate and insular cortices in one or both hemispheres (Figure 2, TFCE corrected, *P*<0.05). For Val/Met subjects, the left middle cingulate cortex (MCC) and IC showed smaller volumes relative to people who were homozygous for the Val allele (TFCE corrected, *P*<0.05; Figure 2).

When grouping Met/Met and Val/Met genotypes as a group of Met carriers, as performed in many prior studies, the Met carriers possessed a smaller GM volume only in the bilateral MCC (TFCE corrected, *P*<0.05; Figure 2).

## Discussion

BDNF is a key regulator of synaptic plasticity. A critical polymorphism at codon 66 of the BDNF amino acid sequence can result in brain anatomy alteration, which was proven by numerous VBM studies carried out mostly in Caucasian subjects [7], [29], [30]. The present study is the first to report patterns of cortical thinning and GM loss in healthy young Chinese adults among BDNF Val66Met genotypes, and the specific role of the Met/Met genotype was evaluated in detail for the first time by combining cortical thickness analysis and VBM, in virtue of a higher frequency of the Met/Met genotype (27.9%) distributed in our present sample. The present study showed that the brain morphology difference in Chinese people carrying the Met allele did display different patterns from Caucasian individuals. In Caucasian people, the difference in brain structure in Met carriers mainly came from Val/Met individuals, because only a few Met/Met subjects were among the Met carriers [11], [31]. However, in our study the Met/Met and Val/Met individuals both showed significant anatomy differences from Val/Val individuals, but the Met carriers (combining Met/Met and Val/Met genotypes) showed the least difference in brain structure. Moreover, the differences in some brain areas were not shared between Caucasians and Chinese, such as the hippocampus which showed a small GM volume in some previous studies based on Caucasians [4], [7], [32] but no difference among BDNF genotypes in our current study. In addition, some regions that were not mentioned in previous Caucasian studies showed anatomical differences among BDNF genotypes in our study. Ethnicity may be a reason for the different effects of BDNF (rs6265) polymorphism in Caucasians and Chinese.

### Brain Structural Difference in Met/Met Genotype Subjects

Following comparison of Met/Met and Val/Val genotypes, consistent differences in cortical thickness and GM volume were found in the present study, particularly in the OFC, MTC, PCC, and IC which showed decreased cortical thickness and GM volume in the Met/Met group. These matching results suggested that these frontal, temporal, cingulate, and insular regions were probably the key areas in which brain structure was determined by BDNF 66Met to some extent in Chinese people.

To date, there has been no study reporting brain structural differences in the BDNF (rs6265) Met/Met genotype alone. The current study was the first to report the effect of the Met/Met genotype on brain morphology using cortical thickness analysis and VBM. Some of our VBM results were consistent with previous studies which were based on Met carriers (combining Val/Met and Met/Met) who had reduced GM volume in the frontal [7], [9], [33] and temporal cortices [11]. Although the Met carriers in these studies were mainly of the Val/Met genotype, together with our VBM and cortical thickness analysis, we believe that the Met allele of BDNF (rs6265) most likely influences brain morphology and that the Met/Met genotype causes the most significant effects, at least in young Chinese adults.

The cortical thickness and VBM analysis also demonstrated GM differences in several other regions of Met/Met subjects, including the superior frontal cortex (SFC), middle frontal cortex (MFC), IPC, SPC, PC, and LOC with reduced cortical thickness, and the IFC, ACC, MCC and ITC with decreased GM volume. The differences in cortical thickness and GM volume in most of these regions were consistent with previous VBM studies [7], [9], [11], [33]excepting the MCC which has never been reported until now. Anatomical studies have showen that the IC has extensive connections with the frontal, parietal, temporal and cingulate cortices [34], [35], [36], [37], which showed reduced GM in our present study no matter the cortical thickness or GM volume. Therefore, this interactive fibers relationship may be directly or indirectly contributed to the GM differences in the MCC, PCC, parietal lobe and IC in Met homozygotes of our present study. However, because VBM and cortical thickness results in some of these regions were not consistent in the present study, the correlation of the BDNF (rs6265) Met/Met genotype and brain anatomical differences in these areas requires further clarification.

### Brain Structural Differences in Val/Met Genotype Subjects

Regarding the comparison of the Val/Met and Val/Val genotypes, matching results were found in the IC by dual-analysis, which were also shown in the comparision between Met/Met and Val/Val groups. These results suggested that this area was most likely influenced by BDNF (rs6265) polymorphism. In addition, decreased GM volume was also found in the MCC in the Val/Met group, and was also observed in the Met/Met group. In addition, reduced cortical thickness in the Val/Met group was found in other areas including the OFC, MFC, SFC, MTC, SPC, PC and LOC which were also included within the cortical thickness decreased regions of the Met/Met group when compared to the Val/Val group. Thus, it can be seen that regions showing differences in Met-heterozygotes overlapped with those of Met-homozygotes. It suggested that substitution of the Met allele for the Val allele in the two homologous chromosomes of the BDNF DNA sequence would result in more severe damage in brain GM. This was demonstracted indirectly by an animal study in which the Met/Met genotype could cause a most significant decrease in the regulated secretion of BDNF by 30% (Val/Met by 18%) [5], where secreted BDNF is known to regulate neuronal development and differentiation. Therefore, extensive decreased GM in Met homozygotes may account for by serious altered neuronal morphology in the present study.

The findings of some previous studies reporting smaller hippocampal volume in Met allele carriers were not replicated in either of our Val/Met or Met/Met subjects. However, our negative results were consistent with several recent reports, in which no evidence for an influence of the BDNF Val66Met genotype on MRI-derived reduced hippocampus volume was found [8], [38], [39], [40]. One reason for these conflicting results may be due to the fact that our study focused on the Chinese population, unlike previous positive studies [6], [7]; however, the genetic effect of BDNF Val66Met polymorphism on hippocampal morphology remains controversial and requires further investigation.

Another point of note is the influence of the grouping method on our results. When we merged the Met/Met and Val/Met groups to obtain the Met carriers group in the VBM analysis, as performed in previous studies [6], [8], [11], [31], the reduction in GM values in brain areas was much smaller in the Met carriers than in the Met/Met group. These results suggested that grouping method of Met carriers may hide some specific roles of Met/Met in brain morphology. To date, we do not know if such a marked discrepancy caused by grouping method also exists in similar analyses based on the Caucasian race because of the low frequency of Met/Met in Caucasians. Nevertheless, our present study has revealed that the function of the Met/Met genotype and Val/Met genotype cannot be represented entirely by a general group of Met carriers. The current findings indicated that availability of a large sample size that can be divided into three genotypes is helpful in clarifying the effect of BDNF Val66Met polymorphism, which should be given more attention in future studies.

### Study Limitations

There are some limitations to our work. The first one is that we didn’t measure the behavioral differences in cognition, emotion, memory and other factors among BDNF Val66Met genotypes. These behaviors related to higher brain function have often been shown to be affected by BDNF Val66Met polymorphism [4], [8], [41]. Thus, if the genetic impact on brain structure in the current study could correlate to behaviors that were close to BDNF function, this would contribute greatly to inferences regarding structural brain differences determined by BDNF Val66Met polymorphism. The second limitation of this study is the small sample size. There were only 15 and 17 individuals in the Met/Met and Val/Val groups respecitvely, which is relatively small for an objective result. Larger sample sizes are required for futher related studies. The third limitation was this study was that comparisons of brain morphology were only carried out at one time-points. To obtain more data, brain morphology data should be collected at two different time –points. These limitations need to be addressed in future related studies.

### Conclusion

In conclusion, the current study demonstrates brain morphology differences related to BNDF Val66Met polymorphism (especially the Met/Met genotype) for the first time in healthy young Chinese adults using a dual analytical method, via cortical thickness analysis and VBM. Unlike Caucasians with significant brain structural differences in Val/Met genotype individuals, Chinese brain anatomy varied mostly in the Met/Met genotype with less GM mainly in the frontal, temporal, cingulate and insular cortices. In addition, by way of analyzing Met carriers, the present study suggested that combining Val/Met and Met/Met genotypes as Met carriers would probably mask the unique role of the Met/Met genotype on brain morphology, which should be taken into account in future studies.

## Materials and Methods

The research procedures were approved by the West China Hospital Subcommittee on Human Studies and were conducted in accordance with the Declaration of Helsinki. All participants were recruited by advertisement, and and gave their written informed consent after they were fully explained the experimental procedure ahead of the experiment. The corresponding content was approved by the West China Hospital Subcommittee.

### Subjects

The data included in this study were drawn from 81 healthy, right-handed Chinese college students (mean age: 20.5±0.88 years, range: 19–23; 48 males). For all subjects, exclusion criteria were: 1) reported history of brain injury; 2) macroscopic brain T2-visible lesions, tumor or obvious cortical/subcortical atrophy on MRI scans; 3) psychiatric disorders evaluated by the Structured Clinical Interviews for DSM-IV Axis I (SCID-I) and Axis II (SCID-II); 4) existence of a neurological disease diagnosed by clinical neurological examination; 5) pregnancy or menstrual period; 6) use of prescription medications within the last month; 7) alcohol, nicotine or drug abuse; 8) dementia. Therer were no differences in IQ among genotype groups according to the Chinese Revised Wechsler Adult Intelligence Scale (WAIS-RC).

### Genotyping

Genomic DNA was isolated from 500 µl of whole blood using TIANamp genomic DNA kit according to the manufacturer’s instructions (TIANGEN BIOTECH CO., LTD., Beijing, China). Polymerase chain reaction (PCR) was carried out in a volume of 20 µLcontaining 10 ng of genomic DNA, 2.5 µL of 10×Taq buffer, 2 µL of dNTP Mixture (2.5 mM), 0.5 µL of Taq DNA Polymerase (2.5 U/µL, TIANGEN BIOTECH CO., LTD.,), 1 µL of each primer and ddH_2_O. The PCRs were done using a BIO-RAD cycler system. The primers were as follows: 5′-AAACATCCGAGGACAAGGTG-3′ (forward), 5′-AGAAGAGGAGGCTCCA AA GG-3′ (reverse). The amplification conditions were initiated at 94°C for 5 min, followed by 30 cycles consisting of denaturation at 94°C for 30 s, annealing at 65°C for 30 s and extension at 72°C for 30 s, with a final extension step of 5 min at 72°C. The PCR products were confirmed by 2% (w/v) gel electrophoresis and purified using the TIANgel Midi purification kit (TIANGEN BIOTECH CO., LTD.). BDNF gene sequence was implemented in Shanghai Bio-tech Co., Ltd. (Shanghai, China). Genotypes of the BDNF Val66Met polymorphism were read by at least two researchers who were blinded to the experiment. For genotypic distribution analysis, the Hardy–Weinberg Equilibrium was tested by the goodness-of-fit χ^2^ test.

### Structural MRI Acquisition

The MRI experiment was performed using a 3.0 Tesla MRI scanner (Magnetom Trio, Siemens) with a standard head coil. A set of T1-weighted high-resolution structural images was collected (TR/TE: 2.7 s/3.39 ms, FOV: 256 mm×256 mm, matrix size: 256×256, flip angle: 12°, in-plane resolution: 1 mm×1 mm, slice thickness: 1 mm with no gaps).

### Cortical Thickness Processing

FreeSurfer 5.0 (http://surfer.nmr.mgh.harvard.edu/) was employed to calculate the cortical thickness from the structural MRI according to the detailed procedures in previous studies [42], [43], [44]. Local cortical thickness was measured on the basis of the difference between the position of equivalent vertices in the pial and gray-white matter surfaces. In brief, cerebral white matter was segmented from the T1-weighted images and the gray-white matter interface was estimated [43]. Topographical defects in the gray-white estimate were fixed, which were then used as the starting point for the deformable surface algorithm search for the pial surface. The surface of the gray-white matter border was inflated, and differences between subjects in the depth of the gyri and sulci were normalized. The reconstructed brain of each subject was deformed and registered to an average spherical surface [44]. To obtain cortical thickness difference maps, the data were smoothed on the surface with a Gaussian smoothing kernel with a full-width half maximum of 10 mm. Statistical thickness difference maps were constructed with *t* statistics (with the inclusion of age and gender as covariates) with a threshold of P<0.05 ((false discovery rate (FDR) corrected)).

### VBM Processsing

Structural data was analysed with FSL-VBM, a voxel-based morphometry style analysis [45], [46] carried out with FSL tools [46]. Structural data were analyzed using FSL-VBM, a voxel-based morphometry style anslysis [45], [46], which was carried out with FSL tools [47]. The structural images were first brain-extracted using BET [48]. Then, the resulting gray matter partial volume images were aligned to MNI152 standard space using the nonlinear registration tool FNIRT, which uses a b-spline representation of the registration warp field. The registered images were averaged to create a study-specific template, to which the native gray matter images were then non-linearly re-registered. The registered images were then modulated (to correct for local expansion or contraction) by dividing by the Jacobian of the warp field. The modulated segmented images were then smoothed with an isotropic Gaussian kernel with a sigma of 3 mm. Finally, group-difference in gray matter volume was tested using permutation-based non-parametric testing (10,000 permutations) with gender and age included as covariates. Multiple comparisons across space were corrected using the threshold-free cluster enhancement method [49]. A difference was considered to be significant at a corrected P<0.05. The minimal number voxels was 8.
